# Priming DNA Replication from Triple Helix Oligonucleotides: Possible Threestranded DNA in DNA Polymerases

**DOI:** 10.4061/2011/562849

**Published:** 2011-09-14

**Authors:** Patrick P. Lestienne

**Affiliations:** U 1053 INSERM, Université Victor Segalen Bordeaux 2, 146 rue Léo Saignat, 33076 Bordeaux, France

## Abstract

Triplex associate with a duplex DNA presenting the same polypurine or polypyrimidine-rich sequence in an antiparallel orientation. So far, triplex forming oligonucleotides (TFOs) are known to inhibit transcription, replication, and to induce mutations. A new property of TFO is reviewed here upon analysis of DNA breakpoint yielding DNA rearrangements; the synthesized sequence of the first direct repeat displays a skewed polypurine- rich sequence. This synthesized sequence can bind the second homologous duplex sequence through the formation of a triple helix, which is able to prime further DNA replication. In these case, the d(G)-rich Triple Helix Primers (THP) bind the homologous strand in a parallel manner, possibly via a RecA-like mechanism. This novel property is shared by all tested DNA polymerases: phage, retrovirus, bacteria, and human. These features may account for illegitimate initiation of replication upon single-strand breakage and annealing to a homologous sequence where priming may occur. Our experiments suggest that DNA polymerases can bind three instead of two polynucleotide strands in their catalytic centre.

## 1. Introduction

Since the pioneering work of Avery et al. is showing that DNA is the support of the genetic information [1] and the characterization of *E. coli* DNA polymerase I by A. Kornberg [2], DNA replication has been the matter of intense researches. 

 Even though, the description of DNA-dependent DNA polymerases seems to completion, the identification of new classes of enzymes, notably repair polymerases, has brought new concepts on DNA integrity, recombination (a major feature providing diversity), and thus on evolution. These proofreading activities may be downregulated leading to mutations and DNA rearrangements causing diseases.

 Basically, replication involves DNA strand separation by DNA helicases, followed by the priming of complementary sequences, or by primase activities yielding Okazaki fragments on the lagging strand. DNA polymerases elongate the 3′ hydroxyl end of the primers on the template strand by phosphodiester bond, resulting from the enzymatic hydrolysis of complementary dNTP into dNMP and the release of pyrophosphate. The free energy resulting from this process is accompanied by a conformational change of the DNA polymerase from an opened to a closed conformation, together with the translocation of the double-stranded DNA.

 Thus, DNA polymerases are known to bind only two DNA strands in their catalytic centre: the template strand and the complementary one under elongation.

 In several cases of diseases, genetics bring the molecular basis of dysfunctions being related with pathology, yielding eventually new concepts in the biochemistry of molecular interactions, notably in DNA transactions mediated through enzymes, among which DNA and RNA polymerases, RNA processing, and their potentially modified properties.

Mitochondrial DNA, by its small size and thus information content, but indispensable one, may offer new insights into novel molecular mechanisms relevant to their downregulated structures; the description of which is thereby presented.

## 2. Skewed Base Composition at Mitochondrial DNA Rearrangement

Studies of mitochondrial DNA breakpoints inducing deletions and even partial tandem triplications, leading to diseases, were first described two decades ago [3, 4] and reviewed in [5]. They revealed that these DNA breakpoints mainly occur between two direct repeats (DRs) [6] separated by several Kb of the 16,6 kb of the human mitochondrial DNA and were localized between the heavy and light strand origins of replication [7]. Analysis of the localization of these heteroplasmic deletions led to conclude that different mechanisms would occur since breakpoints appeared either at perfect repeats or at no repeat, or imperfect direct repeats [6, 8]. The first replicated DR1 was kept at the expense of DR2, and a slippage-mispairing model was proposed by Shoffner et al. involving a nuclease intermediate [9]. Perusal analysis of these DR, from 7 to 13 base pairs, revealed an unexpected information; the nucleotide composition of the synthesized DR1 is skewed in purine nucleotides (A, G) content [10]. This observation reminded us of the work of Felsenfeld et al. [11] who showed by titration that a poly(A) molecule could associate with two poly(U) molecules forming a triple helix. Such unexpected observation, done with a spectrophotometer 4 years after the discovery of the double helix structure, remained somehow on side until researchers found their potential and the general rule governing their formation ([12] for review). The third strand forming the triple helix has a similar sequence (either polypurine or polypyrimidine) than the homologous strand of the base-paired duplex, onto which it binds in an *antiparallel* orientation.

**Figure 1:**
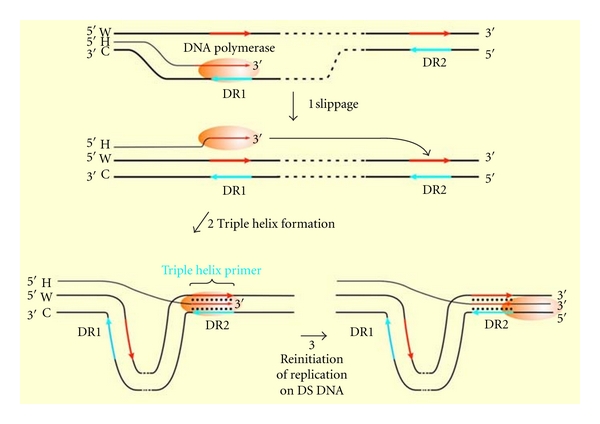
Model for the slippage mispairing between direct repeats. (1) Synthesized purine-rich DR1 sequence the [DNA polymerase-neosynthesized DNA complex] would dissociate from its template and bind to the homologous duplex sequence. (2) The triple helix bound to the duplex DNA is in a parallel orientation as the homologous strand and primes DNA replication on DR2. (3) Elongation of the THP on the DS DNA by DNA polymerase.

The observation that the synthesized DR1 is generally purine rich allowed us to propose that upon dissociation of the (neosynthesized DR1-DNA polymerase complex) and the further binding to the homologous DR2, the tertiary complex could prime and invade the duplex DR2 through the formation of the triple helix, which would be elongated on the double strand (Figure 1). A major issue was the *parallel* orientation required to the triple helix primer (THP) despite the known antiparallel binding of TFO [12, 13].

## 3. Designs of an Enzymatic Assay

To test this hypothesis, initial experiments by gel shift assays showed the association between a neosynthesized purine-rich DR1 of 10 nucleotides long with either a hairpin DNA presenting the binding site of 10-base pairs or the duplex DNA presenting the homologous strand in a parallel orientation. Then, the hairpin DNA containing the double-strand primer binding site was designed such that its 3′ end deoxyguanosine was substituted by a dideoxyguanosine, to prevent its own elongation; its 20 nucleotides-long 5′ end served as a single-strand template. The 5′ end of the potential triple helix primer (10 nucleotides long) was 5′  ^32^P end labelled, so its elongation to 30 nucleotides would provide evidence for its priming property, which was indeed observed when tested with phage T7, T4, the Klenow Fragment, and to a lower extent by Taq and sequenase [14].

Nevertheless, according to the model, the THP had to be elongated on a double-strand that has not a current feature. To test this hypothesis, we used a similar system but with an entire double stranded DNA tethered by thymidine residues to maintain the strand stoichiometry. Results were negative [15]. Then A/A mismatches were introduced nearby the 3′ end of the 10-bases-long primer binding site. Results were positive using T7, Klenow Fragment, and Tth DNA polymerases [15]. Furthermore, a low elongation was evidenced with only one A/A mismatch located even 5 base pairs ahead of the 3′ end of the THP.

**Figure 2:**
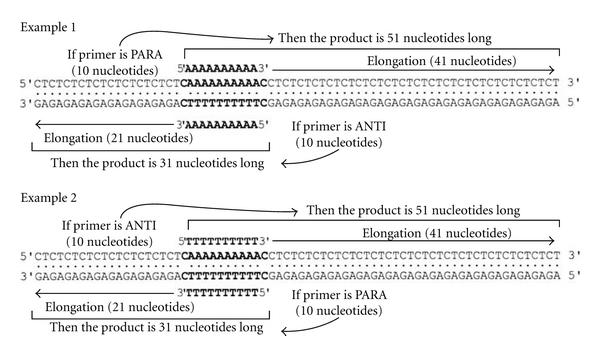
Determination of the orientation of the THP on a double-stranded DNA. The polypurine and polypyrimidine strands (50 pmol) were annealed at equimolar amounts. 5 pmoles of ^32^P-end-labelled primers were tested with their corresponding binding site ending with a C/C mismatch in a replication assay with 5.10–8 M human DNA polymerase *β*. The Reaction media contained either 0.2 mM dGTP, and dATP or 0.2 mM dGTP, dCTP and dTTP. If the primer (A)_10_ was parallel to the homologous strand, then, in the presence of dCTP and dTTP, there is synthesis of a 50 nt-long product. If the orientation was antiparallel, then a 30 nt-long product would be synthesized in the presence of dATP and dGTP. Conversely, if the d(T)_10_ primer is parallel to its homologous primer binding site, then a product of 30 nt-long would be observed in the presence of dGTP, dCTP, and dTTP, while no product was observed with dATP and dGTP [16].

The unusual parallel orientation of the THP was further tested using an orthogonal method by annealing a polypurine strand to a polypyrimidine strand presenting an asymmetric primer binding site ending by a C/C mismatch to allow elongation in both orientations. Depending on the length of the replication product with restricted amounts of dNTP in the assay and the primer used and known to form triple helix [d(A)_10_ or d(T)_10_] (Figure 2), the elongated primer would yield a 50 nucleotides-long product if the lower strand is replicated with the primer d(A)_10_ in a parallel orientation, while it would give a 30 nucleotides-long product or no elongation if the upper strand is the template strand, and thus if the primer is in an antiparallel orientation. Conversely, using d(T)_10_ as a primer, parallel orientation would produce a 30-nucleotides-long fragment while an antiparallel orientation would yield a 50 nucleotides-long product or no elongation [16]. Results from Figure 2 showed a product of about 50 nucleotides-long and thus that the lower strand is replicated from d(A)_10_, while the upper one is replicated with d(T)_10_ as a primer since it provides a 30 nucleotides-long product. These data led us to conclude without ambiguity the parallel orientation of the THP despite their reported antiparallel binding. Other polynucleotides were tested and gave similar results, especially when the pyrimidine primer of 11-residues-long (CTTCTTTCTTC) was used and even at a pH above 8.

These results contrast sharply with previous data showing their *antiparallel* binding and their effect on the inhibition of replication on single strand [17], double strand [18], and even on RNA elongation [19].

## 4. Generalization of These Observations

Since these properties could be restricted to phage and bacterial DNA polymerases, to a single THP 5′d(TGGGGAGGGG)3′, and to binding site 5′d(20 nt..CCCCTCCCCA T4 TGGGGAGGGG..20 nt)3′. complementary nucleotides, but presenting generally A/A mismatches nearby the 3′ end of the primer (DR 64 A) [15], we modified the primer and its binding site of 10 base pairs into a still 8 d(G)-rich primer long with 2 d(A): d(GGGAGGAGGG) (Pur 10). We tested other DNA polymerases, including HIV-1 and AMV reverse transcriptases, human DNA polymerase *β*, *γ* and *λ* as well as Dpo4 from the archaebacteria Sulfolobus *Solfataricus* [20] together with other oligonucleotides known to form triple helices [d(A)_10_, d(T)_10_, d(C)_10_] [16]]. 

**Figure 3:**
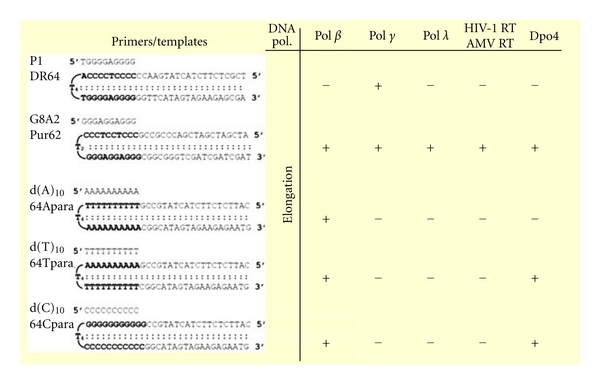
Results of elongation by several DNA polymerases with various templates/primers. Additional templates in an antiparallel orientation (i.e., 3′->5′ instead of 5′->3′) and presenting 3 A/A mismatches were nonproductive as well as 5′d(GGATTACGAG)3′ in a parallel and an antiparallel orientation.

As shown in Figure 3, the repair DNA polymerase *β* elongates all of the tested primers, with the exception of the nonproductive complex with the template: primer 5′d(TGGGGAGGGG)3′ shown in lane 1 (DR 64) but with the notable exception of polymerase *γ*, which, precisely, has to replicate this DR mitochondrial DNA sequence. Furthermore, DR 62C presenting a C/C mismatch is elongated by all tested polymerases. In addition, all polymerases elongate the Pur10 primer with Pur 62 as a template as shown in lane 2. Thus, primers and binding sites allow primer extension, depending on their sequence. However, Dpo4 does not elongate d(A)_10_, and HIV-1 and AMV reverse transcriptases, Pol *γ*, and Pol *λ* are unable to elongate d(A)_10_, d(T)_10_, and d(C)_10_. Thus d(G)A-rich THP/PBS are the most common productive sequences [16]. These results rule out the trivial hypothesis of the opening of the hairpin templates, thus single stranded, followed by the binding of the complementary primer, as a duplex, otherwise all the results would have been similar. However, they indicate differences among DNA polymerases, likely relevant to their unwinding and catalytic properties. Kinetic studies with DNA Pol *β* showed comparable elongation rates for the primer Pur 10 and for a single- or double-stranded template [16]. Therefore, the sequence of the THP and its binding site significantly affect the efficient elongation, even though primers P1 d(TGGGGAGGGG)3′ and Pur10 d(GGGAGGAGGG)3′ display 8 deoxyguanosine residues. This may be due to the peculiar structure of the P1-binding site due to two G4 tracts, as shown by NMR studies.

## 5. Microheterology May Be Tolerated between the Triple Helix Primer and Its Binding Site

**Figure 4:**
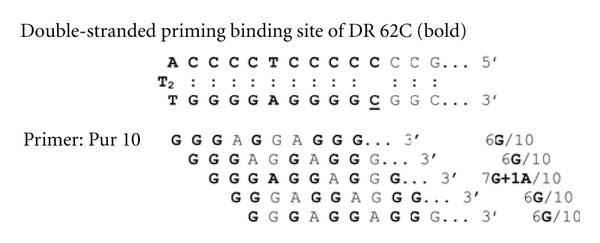
Microheterologies between the primer binding site of DR 62 C, and the 3′ end of primer Pur 40.

To further test the slippage mispairing hypothesis, primer Pur10 length was elongated by 30 nucleotides at its 5′ end (Pur 40) to detect the specificity of its priming property. To this end, elongations on templates Pur 62 and DR 64 as a control were tested, while presenting, or not, a C/C mismatch at position 11 (Pur 62/Pur 62C) and (DR 64, DR 62C), at the theoretical 3′ end of the THP Pur 40. All the tested polymerases elongated Pur 40 with Pur 62 and Pur 62 C as templates in agreement with the previous data. As expected, the control DR 64 was not elongated by any polymerase but by polymerase *γ*. In contrast, using the heterologous template presenting a single C/C mismatch, DR 62C elongation of Pur 40 was evidenced with DNA polymerase *β*, *λ*, HIV-1, and AMV reverse transcriptases and Dpo4. Thus a single C/C mismatch enables elongation of the heterologous primer/primer binding site (PBS). A comparison between the THP and PBS is given on Figure 4. Mismatches of 3 to 4 nucleotides, over 10 nucleotides, of the THP /PBS may still enable DNA elongation; conversely, 6–8 d (G-A) residues at potential similar positions yield productive elongations [16]. These experiments indicate that mismatches between the THP and its PBS may account for rearrangements between imperfect repeats, as pointed before, since as few as 6-7 deoxyguanosine residues may prime DNA replication.

## 6. Biological Implications

During replication, the unwound lagging strand is exposed to DNA breakage by superoxide anions, which, by chance, may occur at polypurine-or polypyrimidine tracts when not protected by single-strand binding proteins. Thus, they could prime a partly homologous double-stranded DNA and invade it. 

The d(G) content is nonrandom in eukaryote genomes, and many G tetrads promote deletions unless peculiar helicases unwind them. d(G) tracts are nonrandomly scattered within the genome but display a skewed representation in oncogene and tumor suppressor genes [21]. Furthermore, guanosine-rich telomeric sequences can stimulate DNA polymerase [22], and d(G)-rich tracts may be cleaved by endonuclease G, yielding 3′OH ends [23]. Triple helices may form either from polypurine/polypyrimidine sequences, H-DNA that are highly recombinogenic [24], together with intramolecular and palindromic structures prone to nuclease cleavage. Furthermore, an increasing number of diseases are associated with DNA forming triple helices [25] and rearrangements, including deletions, duplications, and even triplications, aside the example provided on mitochondrial DNA. The well-established translocation t(14;18) between Bcl2 and IgH inducing follicular lymphoma results from cleavage of one of the 3 dG tracts of the MBR (multiple break region) partly homologous to a sequence of IgH. This sequence forms an intramolecular triple helix with an unpaired strand [26], the complex of which may be cleaved, and the resulting rearrangements have been reported to be partly templated [27]. Z DNA- and non-B-structures are also nuclease sensitive and may be prone to cleavage or recombination [28]. This was even shown in the *E. coli Chi* sequence 5′d(GCTGGTGG) where exonuclease activity stops [29] and might play a role of primer. Furthermore, aside its d(G)-rich sequence, it is similar to the human VTRI, which is highly recombinogenic [30]. In addition, the promoter of the *c-myc* oncogene also displays a triple helix and a G tetrad [31] also found in the telomerase reverse transcriptase activity directed by the RNA template sequence [32]. Perfect or imperfect DR are involved in many cases of DNA rearrangements. Their sequence may slightly differ from one to another, an observation which may be related by tolerating the microheterogeneities shown above, and which may be as short as 6 base pairs long, a very close limit to those encountered in DNA rearrangements yielding diseases. Interestingly, a report showed that DNA invasion occurs before deletion in *Drosophila* [33].

## 7. Possible Shift between the Triple Helix Primer and Its Homologous Strand of the Duplex Binding Site: A RecA Analogy

The mechanisms of recombination involve the first step of synapsis implying recognition of a double strand by a homologous single strand. This reaction is mediated by RecA in *E. coli*, and Rad 51 in eukaryotes. The RecA nucleo-filament recognises a homologous duplex sequence, thus forming a triplex with the homologous strands in a parallel orientation (for review see [34]). Then, the homologous strands are exchanged upon ATP hydrolysis. A structural organization of the base pairings between the double strand and a homologous strand of parallel orientation has been proposed by Zhurkin et al. [35]. Basically the third strand binds to the duplex through bonds with each nucleotide involved in Watson-Crick pairings. This model differs from the Hoogsteen bonds involving recognition of a nucleotide by the complementary one, while in an antiparallel orientation like the homologous nucleotide.

**Figure 5:**
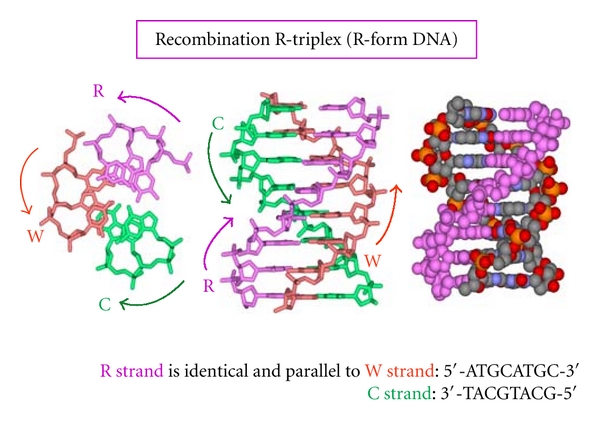
Molecular model of recombination according to Zhurkin et al. [35] (with kind permission). The R strand represents the triple-stranded intermediate in recombination strands mediated by RecA, or THP in our case, while the red molecule seen from the top is the Watson displaced and parallel strand. The middle of the figure shows the same but perpendicular triple helix. The right panel displays the third strand in the triplex conformation.

**Figure 6:**
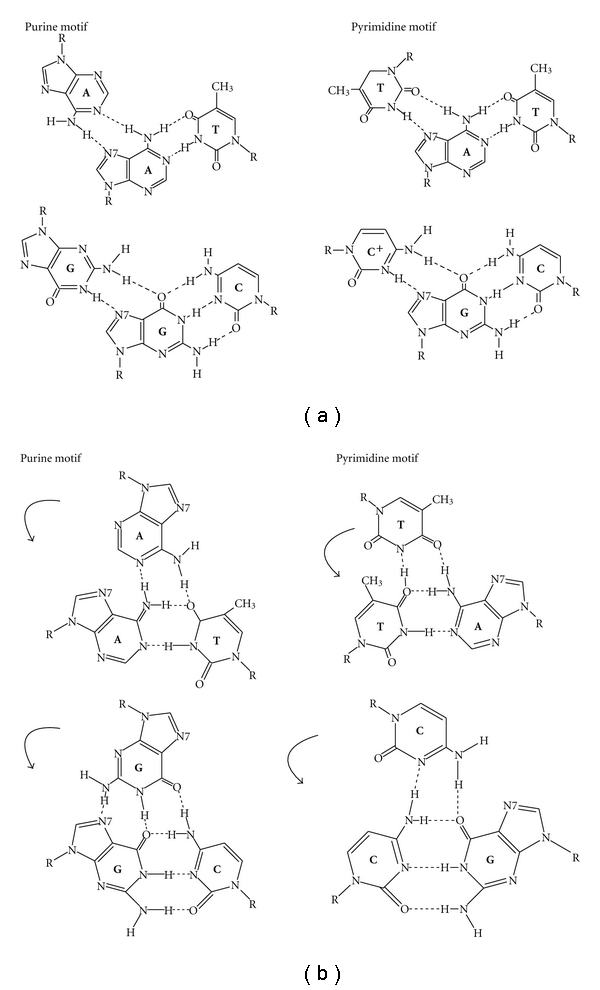
Model of interacting bonds with the R strand, which pairs with Watson-Crick bases modified from Zhurkin et al. [35]. (a) show the classical interactions between triple helix and DS DNA with Hoogsteen and reverse Hoogsteen bonds. Shown in (b) the models could account for the displacement of the homologous Watson strand by the primer or the R strand as in recombination with the invaded duplex without mismatches. The most stable triplexes are G: **G:C**, C: **C:G**, A: **A:T**, and T: **T:A**.

A schematic representation of the pairings described in [35] is shown on Figure 5. Interestingly, the third “parallel” polynucleotide is at about 90 degrees from the homologous one, instead of 180 degrees for the Hoogsteen bonds. The third-strand bound with the Watson-Crick one, in the model of Zhurkin that has been generalized, could account for our results (Figure 6). A recent three dimensional model of the RecA nucleofilament has given high information on this process where the single-strand bond to the RecA polymers displays a B structure able to exchange the DNA strand by stretching the double strand [36] upon the binding of ATP between each monomer.

Moreover, a displacement of 45 degrees towards the homologous nucleotide yields displaced but still similar hydrogen bonds. A further rotation of 45 degrees would then displace the former Watson-Crick bonds. This mechanism requires energy, which, in DNA polymerase, could be brought by dNTP hydrolysis, formation of a phosphodiester bond with the 3′ end of the primer, and conformational change of the DNA polymerase.

## 8. DNA and RNA Polymerases: Catalytic and Structural Comparisons

DNA polymerase I displays a 3′-5′ proofreading and 5′-3′ exonuclease activities. Mild proteolysis yields the Klenow fragment devoid of 3′–5′ activity. The sequence of DNA polymerase I was published [37], its secondary structure predicted [38], and its tertiary structure determined [39]. The replicative phage T7 DNA polymerase was characterized with an unexpected processivity factor and thioredoxin [40] and their three dimensional structure with the template; primer DNA in the catalytic centre was determined [41]. Similarities between DNA polymerases were pointed from their sequences homologies [42] and by their catalytic centre shown by structural studies [43]. They may be compared to a right hand with a palm, a thumb, and fingers. Polymerisation of DNA occurs in the palm between the thumb and the finger by an acid amino acids triad (Asp, Asp, and Glu) and two magnesium ions [44].

Bound to the DNA polymerases, the DNA is generally in the B form but is condensed into the A form during elongation, at least in Taq polymerase [45]. The two bases at the 3′ end of the primer are in the A form with a helical twist and a larger minor groove [45]. A similar transition has been described for complexes with *β* polymerase [46, 47]. Contrasting with the two latter ones, template-primer DNA of T7 DNA polymerase is curved into an S, formed by numerous interactions with the thumb and the fingers. Between 5 to 8 base pairs are away from the catalytic centre [44].

The 5′ end of the template binds the finger's surface. The template/primer is contacted by the finger at the catalytic centre by phosphodiester bonds. The 3′ end of the primer is anchored by the finger and the palm, and is bound to the thumb at its 5′ end. The thumb pushes against the minor groove at 5-6 bases pairs of the catalytic centre with two helices and a loop close to the top of the thumb. Amino acids in the contact of the template strand are localized at the bottom of the helices. DNA polymerase I strand displacement is favoured by the finger subdomains with amino acid residue Arg 84, which interacts with the template, and Ser 769, Phe 771, which favour strand separation [48].

Finally, DNA polymerase I discrimination between rNTP and dNTP occurs during the transition from the opened to the closed conformation of the enzyme [49, 50], while a mutation at amino acid 526 (Tyr→ Phe) has been shown to be critical for the discrimination between NTP to ddNTP [51]. In the eukaryote DNA polymerase *ι*, Hoogsteen base pairings have been discussed [52].

 The RNA polymerases activities, notably of phage T7 [53], are characterized by a pretranslocation [54] and an elongation phase [55]. Several rNMP are added, but the proofreading occurs during this step as RNA polymerase goes forward and reverse on the promoter region formed by an A/T-rich bubble. RNA polymerisation is similar as for DNA polymerases involving an acidic amino acid triad (2 Asp, 1 Glu) and 2 magnesium ions. As for DNA polymerase, the enzyme conformation differs during each nucleotide incorporation. The dissociation of the pyrophosphate upon hydrolysis of the rNTP enables a conformational change, strand separation, and translocation.

The unwinding of the promoter occurs between base pairs −17 to −5. From −4 to 1, there is the formation of the RNA heteroduplex [56] with 2 DNA stands and 1 RNA strand. The RNA: DNA hybrid is in the A form similarly to the last base pairs of the primer: template complex of DNA polymerase. Of interest, a phage T7 RNA polymerase mutant changing Tyr 639 into Phe enables the incorporation of dNTP like in DNA polymerases, reverse transcriptase, DNA- or RNA-directed polymerization depending on the template and the nucleotide in the assay [57]. The elongation phase enables the synthesized RNA to be dissociated from DNA through a tunnel crossing the RNA polymerase. From structural experiments,Steitz et al. concluded to a common mechanism for polynucleotide synthesis by DNA-dependent RNA and DNA polymerases [58] and a convergent catalysis [59].

**Figure 7:**
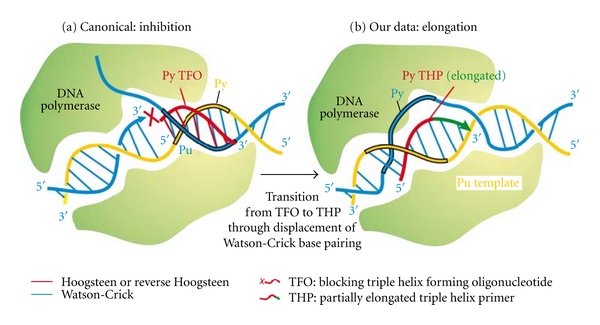
Comparison between the previously documented inhibition of polymerases by triple helices, and interpretation of our data.

 Therefore, our results showing DNA elongation from a triple helix primer by DNA polymerases seem to meet three aspects of RNA and DNA polymerases, as well as of RecA. Actually the triple helix may be the easiest way to accommodate three polynucleotide strands, via a RecA-like activity of DNA polymerases, upon NTP hydrolysis. Of interest to note, the primer is parallel to the homologous strand and may, thereby, as for RecA, displace the homologous strand in the DNA polymerase catalytic centre, base pairs with the template strand, forming thus a transient D-loop with the previous complementary strand (Figure 7). Secondly, DNA polymerases can bind 3 strands, as our results show. However, the way the third strand is dissociated from the double-stranded template is likely to differ from the RNA polymerase tunnel, unless it has not been found. Sequencing experiments revealed a double incorporation of ddNMP facing the transition from purine pyrimidine purine, as if the polymerase or the primer stuttered during elongation [15] or that Tth sequenase incorporated ddNMP facing the nontemplate strand sequence; similarly, termination chain reaction with DNA polymerase *β* showed additional ddNMP incorporation while using a DNA primer, though using an RNA primer, and elongation was as expected [16]. This recalls us the initial pretranslocation complex in RNA polymerases, which stutters before full elongation phase. Finally, RNA and DNA polymerases display the same convergent catalysis mediated by 2 magnesium ions and an acid amino-acids triad. Eventually, computer modelling showed that the third triple helix primer is lying in the major groove of the double-stranded DNA, with the DNA polymerase of phage T7 [14] and Dpo4 [16] catalytic residues close to the 3′ end of the primer.

Further structural experiments may shed lights on this novel property of triple helices and of DNA polymerases under study since five decades.

## Abbreviations

THP: Triple helix primer
PBS: Priming binding site
DR: Direct repeat
Kb: Kilo base.
